# A Systematic Review about Imaging and Histopathological Findings for Detecting and Evaluating Electroporation Based Treatments Response

**DOI:** 10.3390/ijerph18115592

**Published:** 2021-05-24

**Authors:** Vincenza Granata, Roberta Fusco, Simona Salati, Antonella Petrillo, Elio Di Bernardo, Roberta Grassi, Raffaele Palaia, Ginevra Danti, Michelearcangelo La Porta, Matteo Cadossi, Gorana Gašljević, Gregor Sersa, Francesco Izzo

**Affiliations:** 1 Division of Radiology, Istituto Nazionale Tumori IRCCS Fondazione Pascale—IRCCS di Napoli, I-80131 Naples, Italy; v.granata@istitutotumori.na.it (V.G.); a.petrillo@istitutotumori.na.it (A.P.); 2 Oncology Medical and Research & Development Division, IGEA SpA, I-41012 Carpi, Italy; s.salati@igeamedical.com (S.S.); e.dibernardo@igeamedical.com (E.D.B.); m.cadossi@igeamedical.com (M.C.); 3 Radiology Division, Università Degli Studi Della Campania Luigi Vanvitelli, I-80143 Naples, Italy; roberta.grassi89@gmail.com; 4 Italian Society of Medical and Interventional Radiology SIRM, SIRM Foundation, Via della Signora 2, 20122 Milan, Italy; 5 Hepatobiliary Surgical Oncology Division, Istituto Nazionale Tumori IRCCS Fondazione Pascale—IRCCS di Napoli, I-80131 Naples, Italy; r.palaia@istitutotumori.na.it (R.P.); f.izzo@istitutotumori.na.it (F.I.); 6 Radiology Division, Azienda Ospedaliero-Universitaria Careggi, I-50139 Florence, Italy; ginevra.danti@gmail.com; 7 Department of Radiology, UOC San Severo Hospital, 71016 San Severo, Italy; m.laporta.neuro@gmail.com; 8 Department of Pathology, Institute of Oncology Ljubljana, Zaloska cesta 2, SI-1000 Ljubljana, Slovenia; GGasljevic@onko-i.si; 9 Department of Experimental Oncology, Institute of Oncology Ljubljana, Zaloska cesta 2, SI-1000 Ljubljana, Slovenia; gsersa@onko-i.si; 10 Faculty of Health Sciences, University of Ljubljana, Zdravstvena pot 5, SI-1000 Ljubljana, Slovenia

**Keywords:** electrochemotherapy, irreversible electroporation, radiofrequency ablation, imaging findings, histopathological findings

## Abstract

Background: Imaging methods and the most appropriate criteria to be used for detecting and evaluating response to oncological treatments depend on the pathology and anatomical site to be treated and on the treatment to be performed. This document provides a general overview of the main imaging and histopathological findings of electroporation-based treatments (Electrochemotherapy—ECT and Irreversible electroporation—IRE) compared to thermal approach, such as radiofrequency ablation (RFA), in deep-seated cancers with a particular attention to pancreatic and liver cancer. Methods: Numerous electronic datasets were examined: PubMed, Scopus, Web of Science and Google Scholar. The research covered the years from January 1990 to April 2021. All titles and abstracts were analyzed. The inclusion criteria were the following: studies that report imaging or histopathological findings after ablative thermal and not thermal loco-regional treatments (ECT, IRE, RFA) in deep-seated cancers including pancreatic and liver cancer and articles published in the English language. Exclusion criteria were unavailability of full text and congress abstracts or posters and different topic respect to inclusion criteria. Results: 558 potentially relevant references through electronic searches were identified. A total of 38 articles met the inclusion criteria: 20 studies report imaging findings after RFA or ECT or IRE in pancreatic and liver cancer; 17 studies report histopathological findings after RFA or ECT or IRE; 1 study reports both imaging and histopathological findings after RFA or ECT or IRE. Conclusions: Imaging features are related to the type of therapy administrated, to the timing of re-assessment post therapy and to the imaging technique being used to observe the effects. Histological findings after both ECT and IRE show that the treated area becomes necrotic and encapsulated in fibrous tissue, suggesting that the size of the treated lesion cannot be measured as an endpoint to detect response. Moreover, histology frequently reported signs of apoptosis and reduced vital tissue, implying that imaging criteria, which take into account the viability and not the size of the lesion, are more appropriate to evaluate response to treatment.

## 1. Introduction

Several opportunities for cancer treatment include surgery, systemic chemotherapy, radiation therapy and interventional loco-regional procedures. The treatment regimen is linked to the tumor type, stage and several clinical features. Surgical resection when is possible provides the greatest potential for cure. The development of non-surgical, local ablative thermal and non-thermal approaches has provided a new therapeutic option to treat not resectable tumors or to complete tumor eradication.

Radiofrequency ablation (RFA) and microwave ablation (MWA) are the most commonly used techniques, whereas electroporation-based treatments, i.e., electrochemotherapy (ECT) and irreversible electroporation (IRE), have recently emerged as possible alternatives [1,2,3,4].

RFA produces necrosis thanks to thermocoagulation. With RFA, the active tissue heating zone is restricted to a few millimeters nearby to the electrode, with the residue of the target being heated via thermal conduction [5,6]. Consequently, the treatment efficacy is closely related to the lesion size, and the maximum result is obtained for target lesion less than 3.5 cm [5,6]. Additionally, some tissue features, such as electrical conductivity, thermal conductivity, dielectric permittivity and blood perfusion rate, have effect on the RFA procedure efficacy. Especially, RFA treatment should be avoided when the target is near large vessels, due to the heat sink effect [5,6]. However, the bipolar system of the RFA can reduce the heat sink effect and lower pancreatic injury [5,6,7].

MWA used the dielectric effect, which occurs when an imperfect dielectric material is subjected to an alternating electromagnetic (EM) field, generating a larger area of active heating (up to 2 cm close the antenna) allowing more homogeneous necrosis in the target zone, compared to RFA [8,9,10,11]. Moreover, MWA has some improvements with respect to RFA: the target can be greater given that it generates a larger area of necrosis; the treatment time is quicker; and it is less influenced by the defense of the neighboring tissues due to vaporization and charring, so the heat-sink effect impacts less the efficacy of MWA [8,9,10,11].

Conversely, to RFA and MWA, IRE and ECT [12,13,14,15] are non-thermal techniques that cause ablation changing cell membrane permeability thanks to an induced electric field (electroporation). IRE is considered as a direct ablation tool, since electroporation is used in irreversible manner [12,13,14,15]. In fact, several electrodes are placed around the target, using a series of high voltage pulses up to 3000 V and 50 A. These short high-voltage electric fields cause the irreversible permeabilization of the lipid bilayer, the disruption of the cellular homeostasis and the stimulation of apoptotic pathways, causing death of neoplastic cells [12,13,14,15]. IRE can protect surrounding structures, such as the vessels, and it is a central feature when the tumor encases the major vessels [13,14,15]. ECT is conceived on the cells electroporation combined to administration of a single dose of non-permeant or poorly permeant chemotherapeutic agents [16,17,18,19].

Electrical field application to a cell causes a transient and reversible orientation of its polar membrane molecules, with an increase of permeability [16,17,18,19,20]. This transient increase of permeability allows the chemotherapeutic drugs to enter in the cell, thus increasing the cytotoxic effects of the agents. This local potentiation allows increasing the chemotherapy efficacy.

Nowadays, ECT has been incorporated into several cancer treatment guidelines, including cutaneous melanoma, squamous cell carcinoma, breast cancer metastasis or recurrences, Merckel cell and basal cell carcinoma, soft tissue sarcomas and bone metastases [21]. In addition, several studies have evaluated the feasibility and the efficacy of ECT in deep solid tumors such as liver and pancreas both in preclinical and clinical settings [1,22,23,24,25,26].

Ablation therapies have determined a challenge for radiologists, who should assess the treatment response. The locoregional therapy goal is inducing necrosis. Tumor shrinkage may not be apparent or may be absent with thermal loco-regional approaches [6,7,8,9]. ECT is a hybrid treatment where ablation effects are combined with cytotoxic effects [14,20].

The commonly used radiological criteria are based on the cytotoxic effects of the chemotherapeutic agents and follow a change in tumor size as a measure of tumor response [27,28,29,30]. Another aspect that could be linked to tumor response is a decrease of tumor density [31,32]. This change may be linked to cystic change, intratumoral edema or intratumoral hemorrhage. If tumor size and density are increased by hemorrhage (generally in early stage), treatment response interpretation is difficult and may be confused with progression. Therefore, tumor physiologic characteristics such as angiogenesis, water diffusivity and hypoxia are relevant to assess tumor response, and thus, there is need for the development of new functional imaging biomarkers.

A wide spectrum of imaging techniques is available for the evaluation of the outcome of ECT, IRE and RFA: Ultrasound (US), Computed Tomography (CT), Magnetic Resonance Imaging (MRI) and Positron Emission Tomography (PET).

The aim of the manuscript is to provide an overview about the histopathological and imaging findings of electroporation-based treatments compared to thermal approach such as RFA, emphasizing the differences.

## 2. Methods

### Search Criteria

Electronic databases were examined: PubMed (U.S. National Library of Medicine, http://www.ncbi.nlm.nih.gov/pubmed), Scopus (Elsevier, http://www.scopus.com/, accessed on 15 January 2021), Web of Science (Thomson Reuters, http://apps.webofknowledge.com/, accessed on 15 January 2021) and Google Scholar (https://scholar.google.it/, accessed on 15 January 2021).

The following search criteria were used: “radiofrequency ablation” or “reversible electroporation”, or “electrochemotherapy”, or “irreversible electroporation”, and “pancreas”, or “Liver” or “HCC” or “hepatic metastasis”, or “cholangiocarcinoma”.

The research covered the years from January 1990 to April 2021. All titles and abstracts were analyzed.

The inclusion criteria were the following: studies that report imaging or histopathological findings after ablative thermal and not thermal loco-regional treatments (ECT, IRE, RFA) in deep-seated cancers including pancreatic and liver cancer and articles published in the English language.

Exclusion criteria were unavailability of full text, congress abstracts or posters and different topic respect to inclusion criteria.

## 3. Results

We identified 558 theoretically relevant references. After removing 32 duplicates, we obtained 526 references. We identified 64 references through scanning reference lists of the recognized manuscript that we added to the 526 references (total number of articles was 590). We then excluded 345 irrelevant articles and reading abstracts. We salvaged 245 references for further assessment. We excluded 207 references for the reasons itemized in the exclusion criteria. A total of 38 articles met the inclusion criteria: 20 studies report imaging findings after RFA or ECT or IRE in pancreatic and liver cancer; 17 studies report histopathological findings after RFA or ECT or IRE; 1 study reports both imaging and histopathological findings after RFA or ECT or IRE.

### 3.1. Radiological Response Criteria

**Figure 1:**
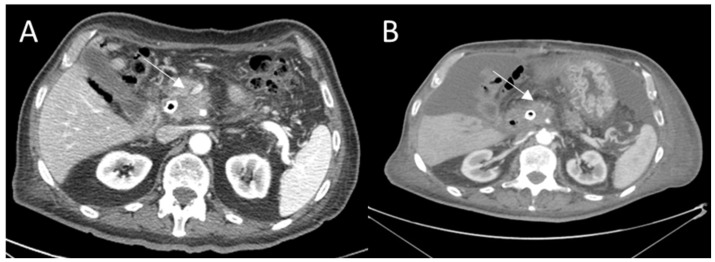
CHOI assessment before (**A**) and after ECT treatment (**B**) of an adenocarcinoma of the pancreatic head. After the treatment in pancreatic phase on CT study, the lesion appears hypodense (**B**, arrow). There was a reduction in lesion density: 36% of reduction in the CT signal before and after treatment (partial response).

The commonly used radiological criteria are based on the chemotherapeutic agents’ cytotoxic effects and are linked to a change in tumor size as a tumor response measure [27,28,29,30]. The WHO (World Health Organization) criteria are based on the concept of assessing tumor burden on the basis of the sum of the products of orthogonal largest diameters of the target lesion (Table 1) while the response evaluation criteria in solid tumors (RECIST) criteria, usually used in most oncology clinical trials, are one-dimensional criteria based on the measurement of the largest diameter of target lesions [28]. However, these criteria only measure the tumor size and do not assess the necrosis, which is the main outcome for evaluating the efficacy of loco-regional therapies. In addition, target therapies could cause increase in tumor size. During treatment, significant changes in tumor density (signal attenuation of Hounsfield unit) measured in CT are observed, due to the tumor necrosis, even though an increase in the lesion size can occur, owing to hemorrhage, necrosis or myxoid degeneration [31,32,33]. The CHOI criteria [32] are CT criteria and evaluate the dimensional changes and/or tumor density attenuation expressed in Hounsfield units (Figure 1, Table 1).

In addition, the European Association for the Study of the Liver (EASL) guidelines have recommended the inclusion of active dose reduction evaluation in the determination of tumor response using imaging techniques with contrast media. Likewise, the AASLD (Association for the Study of Liver Disease) has developed a set of guidelines that modify the RECIST criteria designed to accommodate the concept of vital tumor tissue. These guidelines are known as modified RECIST (mRECIST) [34] and used for the first time in the assessment of hepatocellular carcinoma response. The largest lesion diameter is calculated on the tumor portion that appears “vital” (Table 1) [34]. The viable tumor (Figure 1) can be assessed with protocols including a combination of arterial, portal venous and delayed phase, based on the tumor (e.g., should be measured during the arterial phase for hypervascular tumors and on portal phase for hypovasular tumors) [35,36,37,38,39,40,41,42,43].

Moreover, tumor response may be associated with a decrease in metabolism, without a major reduction in tumor size [44,45,46]. Therefore, metabolic response may be even more predictive of the tumor response than morphologic criteria. PET response criteria in solid tumors (PERCIST) [44] have been proposed to evaluate the fluorodeoxyglucose (FDG) uptake reduction. The response parameter used by PERCIST is the standardized uptake value (SUV), a semi-quantitative measure of activity that represents the tumor activity per weight unity. A reduction of metabolic activity with a SUV decrease represents a treatment response. However, in the literature, “flare response” was reported and linked to uptake increase in responsive cases due to reparative tissue changes after therapy or due to hormone-induced changes in tumor metabolism; this phenomenon can be confused with inflammation that occurs in response to tumor-associated growth factors during progression disease [45,46].

**Figure 2:**
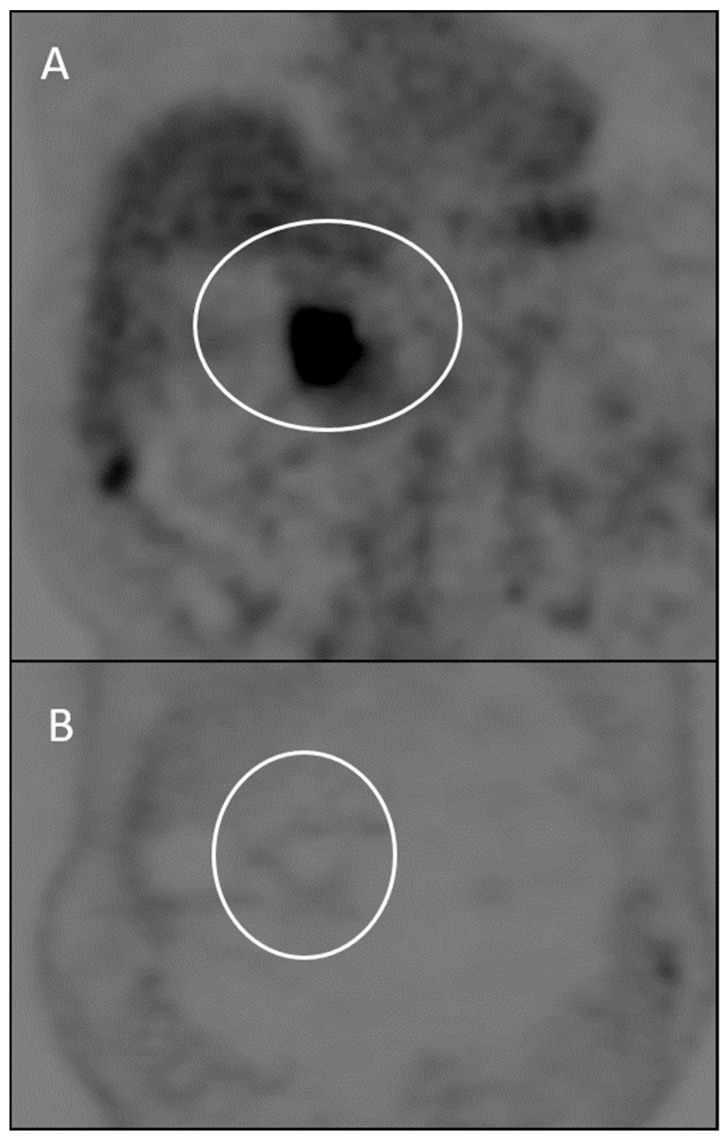
PET/CT images obtained before (**A**) and after (**B**) ECT treatment of locally advanced pancreatic carcinoma. Note complete decline in SUL. This decline represents metabolic complete response by PERCIST.

Anatomical change in tumor size remains an important factor in the PERCIST criteria and should be measured according to RECIST 1.1 (Figure 2, Table 1).

### 3.2. Radiological Tools and Functional Imaging Features

Ultrasound is the most suitable tool for monitoring the immediate tissue response after reversible and irreversible electroporation [47,48,49,50,51]. The safety halo formation around tumor supports the assumption that the tumor was adequately covered by an effective electric field [47,48,49,50,51].

MR and CT were the imaging tools mostly used to assess response to electroporation-based treatments. However, radiological findings post therapy are correlated to the type and method of treatment delivery, to the re-assessment timing and to the used imaging technique.

Several functional parameters [52,53,54] derived from CT and MR have been proposed, which provide data on tissue properties such as perfusion/vascularization and water diffusion. Obtaining this type of data is of utmost importance in electroporation-based treatments, where these parameters properly reflect the rapid and dramatic changes that occur in the tumor microenvironment. Some reports evaluated the suitability of CT and MR outcomes in monitoring and assessing the effects of electroporation treatments [55,56,57,58,59,60,61,62].

CT allows to obtain perfusion imaging while MR allows to obtain both perfusion and diffusion imaging. While perfusion imaging assessed tissue vascularization, diffusion imaging provides information about tissue perfusion, water mobility and cellular density.

Perfusion CT (pCT) can offer images and quantitative hemodynamic parameters based on the relationship between CT enhancement and iodinated contrast agent concentration [63,64]. Several studies evaluated pCT parameters to characterize and evaluate the treatments, demonstrating that pCT could be more appropriate to identify the treatment response in respect to density measurements alone [63,64].

MR imaging was used in order to detect treated regions after ablation procedures in rodent liver tissues [58]: the ablation zones were immediately visible on MR images as hypointense regions on T1-weighted images and as hyperintense regions on T2- and proton density weighted images [58].

Among the different MRI techniques, contrast-enhanced MRI is certainly the most used [63]. Contrast-enhanced MRI with T1-weighted gradient-recalled echo is particularly effective to differentiate treated by untreated area after ablation [55] differentiating the hyperintense enhanced area versus hypointense not enhanced area.

Particularly, dynamic contrast enhanced-MRI (DCE-MRI) allows the quantitative parameters calculation associated to tumor perfusion, vessel permeability and extracellular-extravascular space composition by means of qualitative, semi-quantitative and/or quantitative methods [65].

Microstructural changes in the tumor after electroporation-based treatments result in the removal of barriers to water mobility, this is affected by tumor cellularity, necrosis and other characteristics [65]. Diffusion Weighted Imaging MRI (DWI-MRI) could be used to detect the efficacy of IRE and ECT treatment [61,66,67] being sensitive for the detection of water molecular mobility, cell death and tissue damage [62,65,68,69,70,71]. DWI-MRI data analysis can be done qualitatively and quantitatively, by means of the apparent diffusion coefficient (ADC) calculation using a mono-exponential model. A more sophisticated process for both qualitative and quantitative analysis of DWI-MRI data is the intravoxel incoherent motion (IVIM) method [62,65]. It uses a biexponential model to separately calculate the macroscopic water mobility (contribution to diffusion) and the microscopic blood movement in capillaries (contribution of perfusion). A further method for analyzing DWI-MRI data, known as Diffusion Kurtosis Imaging (DKI) [65], takes into account that water molecules within biologic tissues have a non-Gaussian phenomenon. The idea behind this method is to calculate the kurtosis coefficient (MK), which is associated to the deviance of diffusion phenomenon from a Gaussian approach, and the diffusion coefficient (MD) with the non-Gaussian bias correction.

### 3.3. Radiological Findings on Therapeutic Responses

Imaging interpretations depend highly on the type and method of therapy delivery, on the treatment timing and on imaging technique. An evaluation based only on tumor size is not appropriate to assess the efficacy of such complex treatments, since not always a positive response to treatment is connected to a size decrease; furthermore, dimensional criteria do not allow the differentiation of the fibrosis from the residual tumor.

Table 2 reports main imaging findings after ECT, IRE, RFA in pancreatic and liver cancer.

#### 3.3.1. Pancreatic Treated Lesions

RFA determines heterogeneous appearances on imaging in the ablated areas due to interstitial edema, hemorrhage, carbonization, necrosis and fibrosis as treatment effects. Regarding RFA [72,73,74,75,76,77], the assessment time was between 7 and 34 months and the evaluation was mainly performed by means of CT and MRI.

Rombouts et al. [78] provided a systematic evaluation of the CT findings after RFA in locally advanced pancreatic cancer (LAPC). One week after RFA, the ablation zone appears as a partially sharply defined (83%), heterogeneous area (94%). Three months after RFA, the ablation zone was no longer visible on CT imaging in 67% of patients. Tumor infiltration appears as inhomogeneous enhancement, size increase and circumferential vessel involvement. Additionally, tumor infiltration corresponds to less visibility of ablated area boundaries over time. No necrosis or liquefaction was identified on the CT images. Furthermore, shrinkage of the ablation zone due to amelioration of the inflammatory reaction activated by RFA may also determine a reduction of ablation zone diameter. If the associated edema decreases, a smaller area remains, where the edema shortly after RFA could have been mistaken for an ablation zone part.

According to Paiella et al. for RFA, and in general for “thermal techniques”, the imaging gold standard is CT: a post-ablative hypointense area observed as treatment result [79]. However, also pancreatic tumor is hypointense so that a “qualitative assessment” could cause misdiagnosis. A quantitative evaluation using perfusion or metabolic analysis allows a more objective and correct stratification of patients in responders and non-responders [80,81,82]. A reduction of tissue FDG uptake after ablation is considered an indicator of response while an increase or inadequate decrease of tissue FDG uptake as well as focal or multifocal FDG uptake in the ablation zone margins is highly suggestive of residual or recurrent disease [83]. However, to the best of our knowledge, no papers in the literature discussed main imaging features obtained after RFA or MWA using functional imaging approaches. On the contrary, in the literature, several papers report the evaluation of IRE and ECT efficacy using several functional parameters.

Vroomen et al. [84] assessed IRE findings in LAPC with contrast-enhanced (ce) MRI and ce-CT considering for MRI, the Signal Intensity (SI) on T2-weighted sequences, on T1-weighted sequences (before and after ce, during arterial and venous phase), on DWI and on ADC map; and for CT, the attenuation in the arterial and portal venous phase. Compared to original tumor intensity, one day post-IRE, considering the DWI at b800, MRI signal intensities notably decreased in all cases, accompanied by a subsequent ADC increase. At two and 6-week follow-up, intensity remained low on DWI-b800, in comparison with the initial lesion and high on ADC. One day post-IRE, on precontrast T1-weighted images, small areas of diffuse hyperintensity representing blood residues were visible. The ablation zone contrast enhancement in the arterial and portal venous phase had decreased in all lesions as compared to initial tumor intensity. In the portal venous phase, a hyperintense rim surrounding the IRE ablation zone was found 1 day and 2 weeks post-IRE and was less often identified at 6-week follow-up. At 2- and 6-week follow-up, tumor intensity remained low for the arterial phase and portal venous phase. On the T2-weighted sequences, ablation zone intensity during follow-up did not significantly differ from the initial tumor intensity. However, a remarkable hypointense rim surrounding the ablation zone was observed 2 weeks post-IRE on T2-weighted MRI. Differences between attenuation pre- and post-IRE in the arterial and portal venous phase were not statistically significant on ceCT. Immediately after IRE, intralesional and periablational gas pockets were present in all cases. Post-IRE, the ablation zones were primarily hypodense in the arterial phase after 6 weeks and 3 and 6 months. In the portal venous phase, the ablated areas were slightly hypodense immediately post-IRE; at 6 weeks and 3- and 6-month follow-up, ablation zones were hypodense.

**Figure 3:**
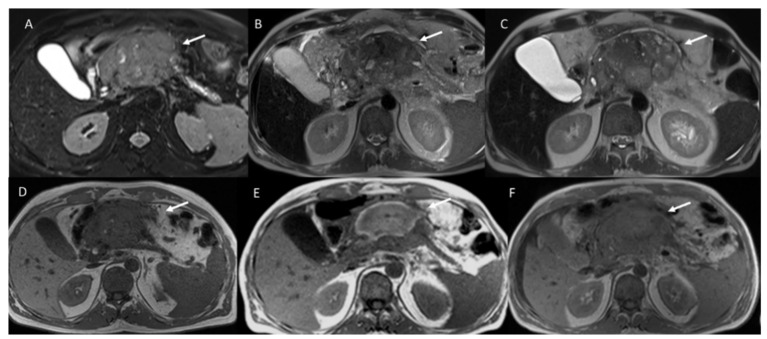
Pancreatic cancer treated with ECT. Pre-treatment, the lesion is hyperintense on T2 weighted sequence (**A**) and hypointense on T1 weighted sequence (**D**). After 1 week, the treated lesion is heterogeneously hyperintense on T1 weighted image (**E**) and heterogeneous hypointense on T2 weighted image (**B**) because of necrosis, hemorrhagic products and dehydration. After 1 month, the imaging appearances change as the blood products evolve and become more homogeneous over time on T2 weighted image (**C**) and T1 weighted image (**F**).

**Figure 4:**
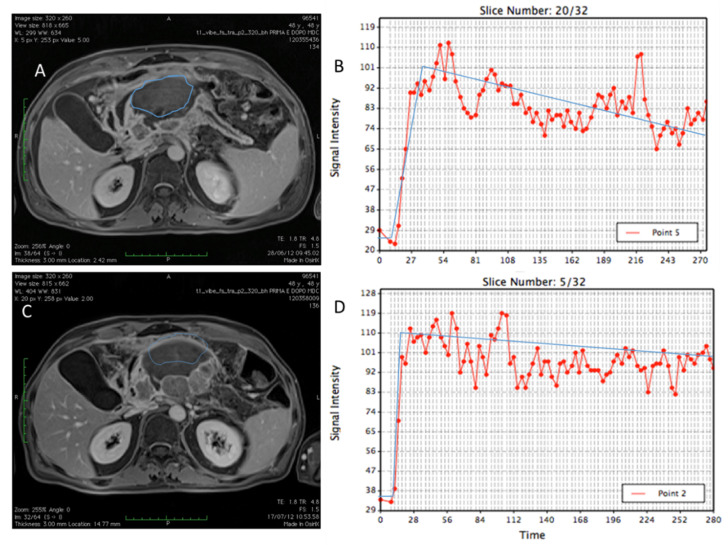
Pancreatic cancer treated with ECT. DCE-MRI pre- (**A**) and post-treatment (**C**) with time intensity curves (**B**,**D**) obtained on segmented region of interest show reduction of lesion enhancement.

**Figure 5:**
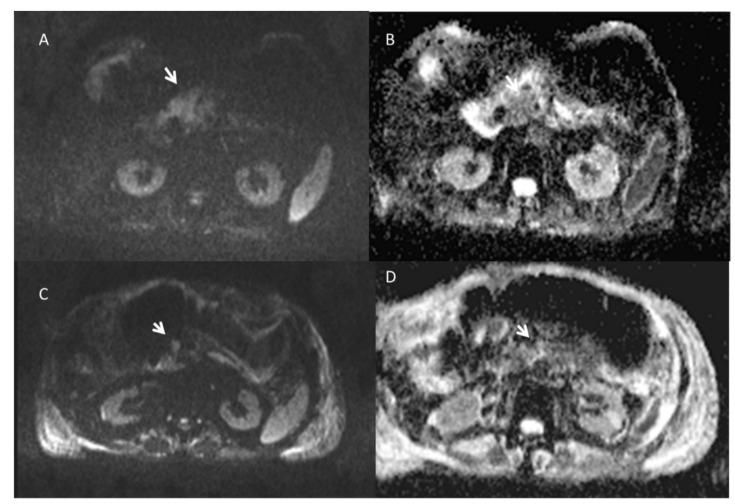
Pancreatic cancer treated with ECT. Pre-treatment, the lesion shows restricted diffusion on b800 s/mm^2^ (**A**) and hypointense SI in ADC map (**B**). After treatment, only vital tumor (arrow) shows restricted diffusion on b800 s/mm^2^ (**C**) and hypointense SI in ADC map (**D**).

Granata et al. [22,26] used morphological and functional parameters to assess the ECT efficacy (Figure 3, Figure 4 and Figure 5) by US, CT, DCE-MRI (calculating wash-in slope (WIS) and wash-out slope (WOS)), DWI (calculating pseudo-diffusivity (Dp), perfusion fraction (fp) and tissue diffusivity (Dt)) and PET. A significant reduction of viable tumor after ECT can be observed according to a significant change of WIS, WOS, Dp and Dt. Perfusion fraction fp showed a significant reduction after ECT only in four patients. No significant difference was observed after ECT in signal intensity of T1-weighted images and T2-weighted images, and in equilibrium-phase of contrast study.

Since necrosis and perfusion modifications, during therapy, often occur before changes in size, DWI may be considered as an early biomarker of the treatment effectiveness [62,65]. Granata et al. in [62] evaluated the diagnostic performance of functional parameters derived by DWI and by DKI. ADC by DWI, MD and MK by DKI were derived. Among investigated diffusion parameters, only the MD showed a significant variation of values between pre and post treatment and a statistically significant difference of percentage change between responders and not responders [62].

#### 3.3.2. Hepatic Treated Lesions

Post RFA ablation areas appear with periablation enhancement due to inflammation in the surrounding parenchyma, and that could depict residual disease gradually decreasing over time [85,86].

**Figure 6:**
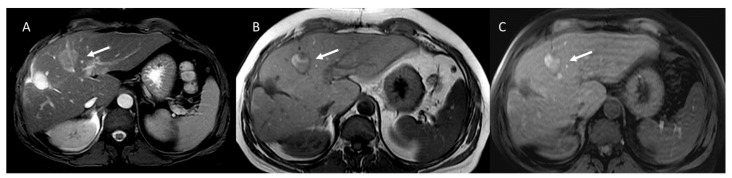
Liver metastasis treated with RFA. The ablated lesion is heterogeneous hyporintense on T2-weighted image (**A**) and heterogeneously hyperintense on T1-weighted images (**B**). On portal phase (**C**), the ablation zone is well demarcated with no enhancement (arrow).

**Figure 7:**
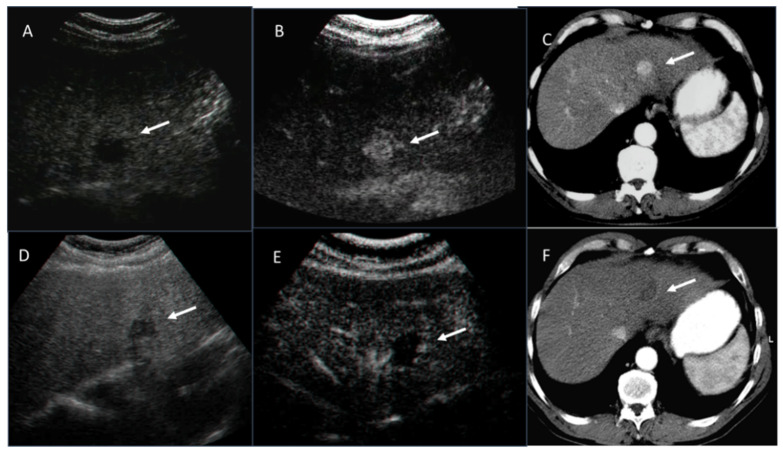
HCC is hypoechoic on US study (**A**) with hyperenhancement during arterial phase in CEUS (**B**) and CT studies (**C**). Post-RFA treatment, the ablated lesion is heterogeneous hypoechoic on US study (**D**) with no enhancement (arrow) during arterial phase in CEUS (**E**) and CT (**F**) studies.

On unenhanced CT performed closely after RFA, the ablation zone is larger than the initial target and is hypoattenuating or heterogeneously hyperattenuating due to coagulative necrosis and hemorrhagic products; however, occasionally, no visible changes may be discernible on CT [85]. At the first follow-up, on ceCT or MRI evaluation, the ablation zone can be spherical, oval, or oblong depending by the number and type of electrodes used. For lesion located between blood vessels, the shape of the ablation zone can be irregular due to “heat sink” effect. On unenhanced CT, the ablation zone becomes more homogeneously hypoattenuating over time. On MRI, the treated lesion is heterogeneously or peripherally hyperintense on T1-weighted images and heterogeneous or hypointense on T2-weighted because of coagulative necrosis, hemorrhagic products and dehydration (Figure 6). Marked hyperintensity on T2-weighted imaging suggests liquefactive necrosis or biloma formation. On ceCT and MRI, the ablation zone is well demarcated, and no enhancement suggests a lack of viable tumor (Figure 7) [85]. Most published descriptions of the contrast imaging findings after RFA for liver tumors have reported that an absence of contrast enhancement is expected within the ablation zone [85].

**Figure 8:**
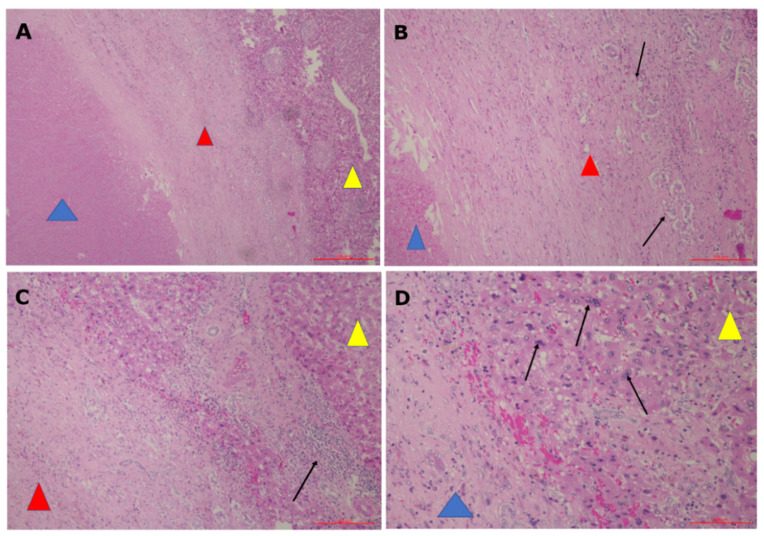
Ablated colorectal liver metastasis and liver parenchyma after ECT (6 weeks after treatment). (**A**) H&E 5×. Blue triangle: necrosis, Red triangle: pseudocapsule, Yellow triangle: liver parenchyma. (**B**) H&E 20×. Blue triangle: necrosis, Red triangle: pseudocapsule, Arrows: bile duct proliferation. (**C**) H&E 10×. Red triangle: pseudocapsule, Yellow triangle: liver parenchyma, Arrow: chronic inflammation. (**D**) H&E 20×. Blue triangle: pseudocapsule, Yellow triangle: liver parenchyma, Arrows: regeneratory changed hepatocytes.

Contrast enhanced US (CEUS) has been employed in the early assessment of the ablated HCC (Figure 8) [87] while the potential role of CEUS in the follow-up of the patient with a successful ablation has not been extensively investigated. Zheng et al. [88] performed CEUS and CT scans and found CT significantly more sensitive in detecting both local tumor progression and new heterotopic lesions. CEUS has the limit of being unable to explore the entire liver during the arterial phase, which is a rather limited time frame; consequently, CEUS could miss a transiently hyperperfused lesion [87].

Contrast-enhanced imaging characteristics of liver tumors that have been successfully treated by IRE ablation differ from those observed after RFA. Specifically, unlike RFA, persistent enhancement of the peritumoral liver parenchyma is observed within the IRE ablation zone, and thus, the ablated margin is not clear in IRE. These are crucial factors for identifying residual tumor [89]. Sugimoto et al. [90] evaluated the diagnostic performance of CEUS, ceCT and ceMRI in the assessment of immediate IRE response in liver lesions. They found, during the arterial phase of CEUS, ce-MRI, and CECT images, five categories of post IRE lesions, according to their enhancement patterns: level 1 lesions showed devascularized areas with or without an ablative margin; level 2 lesions showed linear enhancement within the lesion; level 3 lesions showed rim enhancement; level 4 lesions showed nodular enhancement within the lesion, and level 5 lesions showed enhancement of the entire lesion. At levels 3, 4 or 5, the lesion was considered positive suggestive to incomplete ablation. At levels 1 or 2, the lesion was considered negative suggestive to complete ablation. Continuous real-time observation of hemodynamic changes in ablated lesions is possible with CEUS because microbubbles are pure intravascular tracers that remain in the blood pool. CEUS is, therefore, very sensitive to observe the presence of a residual functional vascular bed in the ablated area and can provide information concerning the vessels remaining in the treated area.

US has been used for observing tissue changes after IRE in a study on pigs [47]. Immediately after the treatment, a hypoechoic area with well-demarcated margins appeared. The rim is possibly attributable to evolving hemorrhagic infiltration via widened sinusoids [47,48]. The hyperechogenic microbubbles that form around the electrodes are a consequence of electrochemical reactions on the electrodes. Around the tumor and in the normal tissue, a hypoechogenic rim is formed, indicating tissue edema [49]. This observation is due to the blockade of the tumor perfusion due to the electric pulse application and water leakage out of the tissue.

Zhou et al. [51] used CEUS to assess the tumor response and characteristics of the ablation zone reporting that immediately after IRE ablation and up to 1 month later, the ablation zones gradually changed from hypo-echogenicity to hyper-echogenicity on conventional US and showed non-enhancement on CEUS.

Sugimoto et al. [90], in a patient with HCC treated by IRE, reported that despite persistent enhancement of peritumoral liver parenchyma within the IRE ablation zone, the tumor itself was clearly demarcated by a devascularized area in comparison to surrounding unablated or ablated liver parenchyma.

Felker et al. [68] reported three distinct layers on MRI in the irreversible electroporation ablation zone: an inner layer of coagulative necrosis (hyperintense at T1- and T2-weighted imaging and non-enhancing), a middle layer of congestion and hemorrhage (hypointense at T1-weighted imaging, hyperintense at T2-weighted imaging and progressively enhancing but hypointense at the hepatobiliary phase) and a peripheral layer of inflammation (hyperintense at the arterial phase but isointense at all other sequences).

In our previous study, we described the MRI findings of HCC treated with IRE at 1-month follow-up [91]. Ablation zones showed a round shape in treated lesions. On T1-weighted images, all lesions showed a nonhomogeneous signal, with a hyperintense central core and a hypointense peripheral rim [91]. On T2-weighted sequences, the signal from the necrotic ablation zone was heterogeneously hypointense [91]. The residual tumor tissue appeared as a peripheral portion that was hypointense on the T1-weighted images and hyperintense on the T2-weighted images [91]. On DWI, treated lesions showed restricted diffusion [91]. The ADC values did not show any statistically significant difference for each single lesion evaluated between baseline and at 1 month, with a large overlap between the ADC values recorded before and after IRE [91]. During the dynamic sequences and the liver-specific phases, the treated area showed signal hypointensity. The ablation zones containing residual viable tumor showed contrast enhancement during the arterial phase and portal phase washout [91]. The residual tumor tissue appeared as hypointense, although to a lesser degree than the necrotic portion, in the hepatobiliary phase. Some lesions showed a transient hepatic intensity differences area within the normal liver parenchyma adjacent to the treated lesions while other had no concentration of liver-specific contrast medium around the ablation zone [91].

Few studies describe the radiological findings of ECT on liver lesions [1,2,3,49,92]: the treated tumor gradually changes into fibrotic tissue during a period of 4 months [93].

Brloznik et al. [92] assessed radiologic findings after ECT on porcine liver by US and CT. Immediately after the end of the treatment, the ablated parenchyma became hypoechogenic, and the hyperechogenic microbubbles started to diffuse. In the next 5–10 min, the hyperechogenic microbubbles diffused through the treated area. After 10 min, the hypoechogenicity started to fade, and in the case of the treatment with hexagonal electrodes, it was no longer visible [92]. The hypoechogenicity of the parenchyma was in contiguity to the treated vessel; however, the vessel wall appeared intact. There was no hemorrhage observed. Doppler examination showed laminar flow [92]. CEUS showed that the perfusion of the treated area was significantly decreased. ceCT at 60–90 min showed subtle hypoattenuating electrode tracks in the pre-contrast and arterial phases, while in the 3 subsequent phases, hypoenhancing areas of treated hepatic parenchyma were noted. No contrast extravasation was identified from large vessels in which the electrodes were inserted [92].

Boc et al. [49] assessed US changes in the liver treated ECT tumors. During the 1st phase (up to 5 min after the delivery of electric pulses), hyperechoic microbubbles were immediately visible along the electrode tracks. During the 2nd phase, after 5–15 min, microbubbles were distributed throughout the treated tumor, and the tumor became hyperechoic and surrounded by a hypoechoic zone [49]. The hyperechoic foci can be an appropriate indicator of the electroporated tissue, and when hyperechoic foci area coincides with the whole tumor mass, this indicated adequate tumor coverage with sufficiently strong electric field. The hypoechoic zone (5–15 mm wide) represents the electroporated area within the normal liver tissue and represents the treatment safety margin [49]. Four days after ECT, US of the metastasis are presented with hyperechoic formation surrounded by a hypoechoic area, which is most likely the edematous area of the liver parenchyma or the safety margin. Five months after ECT, US and MRI showed the metastasis as a fibrotic residuum without the hypoechoic rim. The size of the metastasis was not significantly reduced [49].

Tarantino et al. [2] treated with ECT a prospective case series of patients with liver cirrhosis and Vp3-Vp4-portal vein tumor thrombus (PVTT) from hepatocellular carcinoma (HCC), in order to evaluate the ECT feasibility, safety and efficacy. Post-treatment intraoperative CEUS demonstrated complete absence of enhancement of the thrombosis and of the treated HCC nodule in all cases. In two patients, CEUS showed complete recanalization of the treated PVTT at 2 and 3 mo follow-up, respectively. In these two patients, CEUS and CT confirmed complete patency of the vessel without any intravascular or perivascular recurrence during follow-up. In three patients, CT and CEUS showed permanent complete thrombosis with a persistent, shrunk, avascular thrombus into the treated vessels. In all three cases, no intravascular or perivascular enhancement consistent with residual tumor or local recurrence was detected at CT and CEUS during follow-up. In the remaining patient, 24 h post-treatment CEUS showed absence of enhancement of the treated thrombus.

**Table 2:** Main imaging findings after ECT, IRE, RFA in pancreatic and liver cancer.

Imaging Modality	Cancer Type	ECT	IRE	RFA	References
Ultrasound	pancreatic	Around the tumor and in the normal tissue, a hypoechogenic rim is formed.	Immediately after the treatment, a hypoechoic area with well-demarcated margins appeared. The rim is possibly attributable to evolving hemorrhagic infiltration via widened sinusoids.	CEUS cannot substitute CT or MRI in post-ablation RFA follow-up	[22,26,47]
			Hyperechoic foci can be indicators of the electroporated tissue and when their area coincides with the whole tumor mass a complete lesion coverage can be considered.		
Magnetic Resonance Imaging		No significant difference was observed after ECT in signal intensity of T1-weighted images and T2-weighted images and in equilibrium-phase of contrast study.	Hypointense regions on T1-weighted images and hyperintense regions on T2-weighted images are visible. Small areas of diffuse hyperintensity representing blood residues were detected in all ablated areas on precontrast T1-weighted images.	At the best of our knowledge no papers in literature reported imaging features on efficacy of ablation by RFA using MRI functional approaches.	[22,26,62,65,79,84]
		Electrodes were visualized as signal voids on gradient recalled echo images, resulting in larger signal voids than those depicted on Turbo Spin Echo images.	Electrodes were visualized as signal voids on gradient recalled echo images, resulting in larger signal voids than those depicted on Turbo Spin Echo images.		
		Perfusion fraction fp by DWI showed a significant reduction after ECT.	In the portal venous phase, 1 day and 2 weeks post-IRE, hyperintense rim surrounding the ablation zone. At 2- and 6-week follow-up, tumor intensity remained low for the arterial phase and portal venous phase		
		The MD derived by DKI showed a significant increase between pre and post treatment and a significant statistically difference for percentage change between responders and not responders.	Post-IRE DW-MRI signal intensities notably decreased accompanied by a subsequent ADC increase.		
Computed Tomography		No significant difference was observed after ECT in CT equilibrium-phase of contrast study.	Immediately after IRE, intralesional and periablational gas pockets	One week after RFA, the ablation zone was visible as partially sharply defined and heterogeneous area	[22,26,78,79,84]
		Reduction of HU density was observed on treated area.	Post-IRE the ablation zones were primarily hypodense in the arterial phase after 6 weeks and 3 and 6 months. In the portal venous phase, the ablated areas were slightly hypodense immediately post-IRE; at 6 weeks and 3- and 6-month follow-up	hypointense area and inhomogeneous enhancement are observed as result of the treatment.	
PET/CT		A reduction of FDG uptake in the lesion after ablation is indicator of response while an increase or inadequate decrease of FDG uptake in the lesion after ablation, as well as focal or multifocal FDG uptake in the margins of the ablation zone, is highly indicative of residual or recurrent disease.			[22,26,83]
Ultrasound	liver	Hyperechoic microbubbles were observed along the electrode tracks. Then 10–15 min, microbubbles were distributed throughout the treated tumor, and the tumor became hyperechoic and surrounded by a hypoechoic zone. The hyperechoic foci can be appropriate indicators of the electroporated tissue and when their area coincides with the whole tumor mass, they indicated adequate tumor coverage with sufficiently strong electric field. CEUS showed that the perfusion of the treated area was significantly decreased or that there was a complete absence of enhancement for responsive area. Responsive area 5 months after the treatment is observed as fibrotic residuum without the hypoechoic rim.	The ablation zones gradually changed from hypo-echogenicity to hyper-echogenicity on conventional US and showed non-enhancement on CEUS. On CEUS treated responsive areas are visible as devascularized areas with or without an ablative margin or linear enhancement within the lesion. Continuous real-time observation of hemodynamic changes in ablated lesions is possible with CEUS because microbubbles are pure intravascular tracers that remain in the blood pool.	Post RFA treatment the ablated responsive lesion is heterogeneous hypoechoic on US study with no enhancement during arterial phase in CEUS. CEUS may miss a transiently hyperperfused lesion just because not exploring that given liver area.	[2,3,47,48,49,51,87,88,89,90,92]
Magnetic Resonance Imaging		Persistent enhancement of the peritumoral liver parenchyma is observed within the IRE ablation zone.	On T1-weighted images, treated responsive lesions showed a nonhomogeneous signal, with a hyperintense central core and a hypointense peripheral rim. On T2-weighted sequences, the signal from the necrotic ablation zone was heterogeneously hypointense. The residual tumor tissue appeared as a peripheral portion that was hypointense on the T1-weighted images and hyperintense on the T2-weighted images. Three distinct layers on MRI in the irreversible electroporation ablation zone are visible: an inner layer of coagulative necrosis (hyperintense at T1- and T2-weighted imaging and non-enhancing), a middle layer of congestion and hemorrhage (hypointense at T1-weighted imaging, hyperintense at T2-weighted imaging, and progressively enhancing but hypointense at the hepatobiliary phase), and a peripheral layer of inflammation (hyperintense at the arterial phase but isointense at all other sequences).	On ceMRI spherical, oval or oblong area dependent to the number and type of electrodes used are visible. Treated lesion is heterogeneously or peripherally hyperintense on T1-weighted images and heterogeneous or hypointense on T2-weighted because of coagulative necrosis, hemorrhagic products and dehydration. The ablation zone is well demarcated, and no enhancement suggests a lack of viable tumor	[68,85,87,88,89,90,91]
		Responsive area 5 months after the treatment is observed as fibrotic residuum without the hypointense peripheral rim.	On ceMRI treated responsive area are visible as devascularized areas with or without an ablative margin or linear enhancement within the lesion. The ablation zones containing residual viable tumor showed contrast enhancement during the arterial phase and portal phase washout. The residual tumor tissue appeared as hypointense, although to a lesser degree than the necrotic portion, in the hepatobiliary phase		
		On ceMRI, no intravascular or perivascular enhancement was observed in responsive area.	On DWI, treated lesions showed restricted diffusion.		
Computed Tomography		ceCT showed subtle hypoattenuating electrode tracks in the pre-contrast and arterial phases, while in the 3 subsequent phases, hypoenhancing areas of treated hepatic parenchyma were noted. No intravascular or perivascular enhancement was observed in responsive area.	On ceCT treated responsive area are visible as devascularized areas with or without an ablative margin or linear enhancement within the lesion	Periablation enhancement occurs as a result of inflammation in the surrounding parenchyma. Hypoattenuating or heterogeneously hyperattenuating is visible as coagulative necrosis and hemorrhagic indicators.	[2,3,85,87,88,89,90,92]
		Responsive area 5 months after the treatment is observed as fibrotic residuum without the hypointense peripheral rim.	Persistent enhancement of peritumoral liver parenchyma within the ablation zone; the tumor itself was clearly demarcated by a devascularized area in comparison to surrounding unablated or ablated liver parenchyma	On ceCT the ablation zone is well demarcated, and no enhancement suggests a lack of viable tumor. Spherical, oval or oblong area dependent to the number and type of electrodes used are visible	
PET/CT		A reduction of FDG uptake in the lesion after ablation is indicator of response while an increase or inadequate decrease FDG uptake in the lesion after ablation, as well as focal or multifocal FDG uptake in the margins of the ablation zone, is highly indicative of residual or recurrent disease.			[83]

Note. CT: Computed Tomography; ceCT: contrast enhanced CT; MRI: Magnetic Resonance Imaging; ceMRI: contrast enhanced MRI; DWI: diffusion weighted imaging; CEUS: contrast enhanced ultrasound; US: ultrasound; ECT: electrochemotherapy; IRE: irreversible electroporation; RFA: radiofrequency ablation; DKI: diffusion kurtosis imaging; MD: mean diffusion coefficient; FDG: fluorodeoxyglucose.

### 3.4. Histopathological Findings after Electroporation Based Treatments

The imaging results are in agreement with the histopathological findings on tumor nodules successfully treated with thermal ablative techniques, such as RFA and MWA, showing several features of coagulation necrosis of the hepatic parenchyma. Ablative treatments cause almost instantaneous protein coagulation with irreversible damage to mitochondria and cytosolic cell enzymes, ultimately leading to carbonization [94]. Macroscopically, the RFA-ablated zone in the liver appears as a localized hardening of the hepatic parenchyma, with a central white area and peripheral red zones [95]. In the acute stage, vital stains demonstrate loss of vitality immediately after ablative treatments. Electron microscopy and TUNEL staining showed destroyed nuclei and mitochondria, and histochemical evaluation showed absence of cell viability markers confirming irreversible cell damage [96].

Four weeks after treatment (chronic stage), the necrotized tissue undergoes volume loss and becomes stone-hard, surrounded by a fibrous capsule. In the chronic stage, hematoxylin-eosin staining shows cytolysis, amorphous eosinophilic hepatocyte and mononuclear cell infiltration. The liver tissue specimens show complete tissue necrosis [97] surrounded by a hypocellular fibrous rim [94,98]. Histopathological findings in liver specimens after ablative treatments support the typical computed tomography (CT) or magnetic resonance (MR) imaging results describing the ablated zone as a round or oval area showing defect in contrast enhancement. The size of the ablated zone in the liver gradually decreases over time, showing a mean percentage volume change equal to 79% one month after treatment [99].

Regarding non-thermal ablative techniques, several studies investigated tissue histology after ECT in different tumor types. The histopathological findings on tumor nodules successfully treated with ECT are supported by the mechanism of action of ECT. The effect of ECT on tumor tissue is due to a combination of different components: (i) the direct cell damage caused by the cytotoxic activity of bleomycin; (ii) the indirect damage by vascular effects and (iii) immuno-inflammatory reactions.

In cutaneous melanoma metastases, early signs of epidermal degeneration, an increase of the inflammatory infiltrate, and initial tumor cell morphological changes can be detected as early as 10 min after ECT. The cell damage increased progressively during the following days, reaching a climax 3 days after treatment and continuing until 10 days. Scarring fibrosis and complete absence of tumor cells were observed at two months after treatment. The observations suggest both a direct necrotic cell damage and a rapid activation of apoptotic mechanisms that occur in the early phases of the cutaneous reaction to ECT [100].

In breast cancer, histological images collected one month after ECT showed necrosis of the target tissue and different areas with marked fibrosis and entrapped foamy, histiocytes and chronic inflammatory cells [101]. In basal cell carcinoma, histology performed after ECT showed no tumor remnant, diffuse fibrosis and several empty pseudocystic spaces as a result of tumor destruction caused by ECT treatment [102].

In agreement with these findings, in metastatic spine melanoma, post-ECT biopsy showed intertrabecular fibrosis and tissue necrosis with focal residual nests of viable tumor cells. The overall percentage of necrosis reported after ECT was greater than 95% [103]. The effect of electroporation on bone has been extensively studied in animal models: Tschon et al. [104] investigated the feasibility and safety of delivering electrical pulses on healthy bone and structures of clinical relevance. The authors showed that irreversible electroporation removed cells from trabeculae in the femurs of rabbits and in the vertebral body of sheep. After irreversible protocol, microtomography demonstrated that the trabecular structure was maintained while histological analysis showed the presence of new bone marrow cells, osteoblasts and mineral apposition characterized by new trabeculae thinner than controls (*p* = 0.005). In rabbit femoral bone, Fini et al. evaluated the electrical field sufficient to ablate bone cells in the targeted area [105]. The authors reported that the ablation area, measured 7 days after treatment, increases in parallel with the number of pulses and/or with the amplitude of the electrical field applied. After IRE protocol, histology showed no tetracycline labelling among the electrodes, and ablation of osteoblasts from the trabeculae. Thirty days after treatment, tetracycline labelling showed deposition of new bone among the electrodes and histological analysis showed marked remodeling activity. In a rat model of induced bone metastases, Fini et al. [106] reported that trabecular bone tissue was almost completely replaced by tumor tissue and bone marrow spaces were infiltrated by malignant tumor. After ECT treatment, inhibition of bone metastasis development was observed. Bone structure was well preserved, there was no loss of bone density or organization, and trabecular density was not compromised. Moreover, similarly to the healthy control group, ECT-treated animals showed intense osteoblast activity on the trabecular surface displaying new bone formation. These data demonstrate that ECT acts specifically on the tumor cells while leaving healthy surrounding tissue unharmed and preserving bone structure and trabecular density. Moreover, tissue regeneration following EP-based ablative treatments can explain the “flare response” in PET scans linked to increased FDG uptake reported in responsive patients [45,46]. In fact, being FDG uptake not tumor specific, it can also be seen in healthy tissue as inflammation or post-traumatic repair and could be mistaken for tumor recurrence.

In preclinical studies performed on healthy porcine liver, Brloznik et al. [92] reported no clinically significant damage to the liver after ECT. Radiologic examinations of the treated areas showed intact vessel walls and patency. In particular, the authors noted no difference between electroporation alone and ECT, confirming that bleomycin at the dosage used has a negligible effect on healthy tissue. In agreement with these findings, Zmuc et al. [107] reported the results of histological examination at different time points after ECT. Analysis at 7 days after ECT, showed areas of coagulation necrosis and areas of fibrotic proliferation. Fibrotic areas were surrounded by intact liver parenchyma with regenerative marginal hepatocytes. Large blood vessels and bile ducts were largely unaffected by ECT, suggesting the preservation of the collagen matrix scaffolding of vessels. Similar areas of coagulation necrosis, sinusoidal congestion with relative preservation of microvasculature, and inflammatory cell infiltration have also been described after IRE in porcine liver [57]. However, compared to ECT, IRE causes irreversible damage to both healthy and tumor cells within the ablated area, and thus, the expected degree of fibrosis in the long term after IRE treatment might be greater than after ECT.

In patients affected by colorectal liver metastasis, Gasljevic et al. [93] reported coagulation necrosis in the treated area encompassing both tumor and a narrow area of normal tissue. The ECT-treated area became necrotic and encapsulated in a fibrous tissue (Figure 8). The functionality of most of the vessels larger than 5 mm in diameter and a large proportion of biliary structures were preserved while smaller blood vessels showed different degrees of damage. Few signs of vascular damage, limited to the endothelial cells, have been also reported by Bigi et al. [100] in melanoma skin metastasis. Worth of notice, Gasljevic et al. [93] also reported regenerative changes in peripheral parts of the targeted liver parenchyma already 8–10 wks after ECT (Figure 8).

In patients affected by metachronous colorectal liver metastasis undergoing two-stage surgical resection, histological analysis of the ECT treated metastasis was available at 6–12 weeks after treatment. The histology of resected metastases treated by ECT showed significant lower percentage of vital tumor tissue (*p* = 0.001) compared to non-treated ones. These results further support the hypothesis that imaging techniques aimed at evaluating tumor viability more than lesion size would be more accurate to evaluate response to ECT in deep-seated tumors [108].

Table 3 reports the main histopathological findings of ECT, IRE and RFA.

## 4. Discussion

Assessment of tumor response to oncological treatments is difficult, especially when ablative techniques are used. Conventional criteria based only on the lesion size have shown to be not adequately appropriate at assessing the treatment efficacy. Additional functional data has proved to reveal hidden information about the tumor status after ablative treatments.

Imaging features are related to the type of therapy administrated, to the timing of re-assessment post therapy and to the imaging technique being used to observe the effects. A qualitative assessment based on the images’ evaluation performed by the radiologist could overcome the difficulties related to the abovementioned differences due to different imaging techniques; however, human error factors are extremely likely and would cause misclassification and misdiagnosis. On the other hand, a quantitative evaluation, based on perfusion evaluation or metabolic analysis, allows a more objective reassessment and a more correct stratification of patients in responders and non-responders. However, this approach requires criteria to be standardized, so that different data from different imaging techniques can be consistently compared.

This was specifically addressed by Granata et al. [26], which considered ECT treatments in locally advanced pancreatic cancer patients. Their results confirmed that the use of purely dimensional morphological criteria (RECIST 1.1 and mRECIST) in the evaluation of solid tumor response to ECT is inadequate, whether they are obtained from CT or MR imaging. On the contrary, the authors found that, in patients evaluated with CHOI criteria, PERCIST criteria and with the perfusion and diffusion parameters derived from MR imaging, it was possible to predict the partial response, the stable disease and disease progression status. Similar differences, although not statistically significant, were also reported by Campanacci et al. (in press on Eur J of Surgical Oncology) after ECT treatment of bone metastases.

Edhemovic et al. [108], in a prospective phase II study including patients with metachronous colorectal liver metastases, assessed the response to ECT using mRECIST, demonstrating an objective response of 75% (63% CR, 12% PR) and a median duration of the response of 20.8 months for metastases in CR and 9.8 months for metastases in PR.

Djokic et al. [23] treated 17 hepatocellular carcinomas with ECT, reporting a complete response rate at 3–6 months of 80% per patient and 88% per treated lesion. Lesions treated in this study were assessed by contrast-enhanced CT or MRI using a distinct hepatocyte contrast; the images were evaluated using CHOI criteria to assess the treatment response.

However, while mRECIST, CHOI and PERCIST are standardized criteria, perfusion and diffusion imaging parameters still measure the percentage difference calculated from the baseline; as a consequence, the treatment response category is defined by appropriate thresholds, generally calculated using ROC analysis on single-patient datasets. Moreover, these criteria require proper images post-processing that are not yet implemented on commercially available workstations and that, therefore, cannot be used in clinical practice.

Moreover, as reported by Mali et al. [109] and by Sersa et al. [110], the difference in the ECT effectiveness is linked to tumor histotype. ECT demonstrated higher activity in superficial sarcomas (OR 99.3%, CR 73.9%) compared with carcinomas (OR 81.1%, CR 62.7%) or melanoma (OR 80.6%, CR 56.8%). In contrast, the multi-institutional IMI-GIDO study by the Italian Melanoma Intergroup did not detect any significant differences in response among histotypes [111]. However, the issue of the tumor response is complex because it can depend by different factors (e.g., tumor size, anatomical location, and exposure to previous oncologic treatments), which may directly or indirectly influence the final effect. Several ECT clinical studies have observed significant differences in tumor response according to the treated lesions size [110]: lesions smaller than 3 cm in size, either superficial or deep-seated, exhibit a higher response rate than larger lesions. Instead, Mali et al. [109] proposed a 2 cm cut-off, which was shown to be associated with the most significant complete response. More recently, an analysis of the InspECT registry (2482 lesions in 987 patients) [112] documented a steady progressive decrease in the complete response with the increase in tumor size, with rates consistently higher than 70% for tumors up to 1.5 cm in size. Recent evidence indicates that preirradiated tumors have a significantly lower response rate to ECT [110].

A better response to ECT was ascribed to better perfusion of the carcinoma, and tumor vasculature was proposed as a predictor of the response to ECT [110]. For example, colorectal liver metastases are less vascularized than hepatocellular carcinoma and are less responsive to ECT [110].

In addition, a dense and aberrant extracellular matrix affects drug diffusion and pharmacokinetics, contributing to insufficient or heterogeneous drug distribution in solid tumors [110].

## 5. Conclusions

Altogether, the histological findings after both ECT and IRE show that the treated area becomes necrotic and encapsulated in fibrous tissue, suggesting that the size of the treated lesion cannot be measured as an endpoint to detect response. Moreover, histology frequently reported signs of apoptosis and reduced vital tissue, implying that imaging criteria, which take into account the viability and not the size of the lesion, are more appropriate to evaluate response to treatment.

## Figures and Tables

**Table 1:** Comparison of WHO, RECIST 1.1, Choi, mRECIST, PERCIST Criteria.

Response	WHO	RECIST 1.1	CHOI	mRECIST	PERCIST
Complete response	No lesions detected for at least 4 weeks	Disappearance of all target lesions or lymph nodes < 10 mm in the short axis	Disappearance of all target lesions	Disappearance of arterial phase enhancement in all target lesions	Disappearance of all metabolically active tumors
Partial response	≥50% decrease in SPD (confirmed at 4 weeks)	>30% decrease in SLD of target lesions	≥10% decrease in tumor size or ≥15% decrease in tumor attenuation at CT; no new lesions	>30% decrease in SLD of “viable” target lesion (arterial phase enhancement)	>30% (0.8-unit) decline in SUL peak between the most intense lesion before treatment and the most intense lesion after treatment
Progressive disease	≥25% increase in SPD in one or more lesions; new lesions	>20% increase in SLD of target lesions with an absolute increase of ≥5 mm; new lesions	≥10% increase in SLD of lesions; does not meet the criteria for partial response by virtue of tumor attenuation, new intratumoral nodules or an increase in the size of the existing intratumoral nodules	>20% increase in SLD of “viable” target lesion (arterialphase enhancement)	>30% (0.8-unit) increase in SUL peak or confirmed new lesions
Stable disease	None of the above	None of the above	None of the above	None of the above	None of the above

Note. CT: Computed Tomography; WHO: World Health Organization; RECIST: response evaluation criteria in solid tumors; mRECIST: modified RECIST; PERCIST: PET response criteria in solid tumors; SPD: sum of products of diameters; SLD: sum of longest diameters; SUL: standardized uptake value corrected for lean body mass.

**Table 3:** Histopathological findings of ECT, IRE and RFA.

	ECT	IRE	RFA/MWA	Reference
Inflammatory infiltrate	✓		✓	[100,101]
Fibrosis	✓	✓	✓	[94,98,100,101,103]
Necrosis	✓	✓	✓	[100,101,102,103]
Tissue Regeneration	✓	✓		[93,107]
Apoptosis	✓	✓		[100]
New tissue formation	✓	✓		[105,106]
Preservation of collagen matrix	✓	✓		[57,107]
Preservation of blood vessels larger than 5 mm	✓	✓		[93,107]
Preservation of biliary structures	✓	✓		[93,107]
Damage to small vessels (limited to endothelial cells)	✓	✓		[93,100]
Carbonization			✓	[94]
Destroyed nuclei and mitochondria			✓	[96]
Viability markers	Reduced	Absent	Absent	[93,96,107]

Note: ECT: electrochemotherapy; IRE: irreversible electroporation; RFA: radiofrequency ablation; MWA: microwave ablation.

## Data Availability

All data are reported in the manuscript.
